# Comparison between Constrained and Semiconstrained Knee Allograft-Prosthesis Composite Reconstructions

**DOI:** 10.1155/2013/489652

**Published:** 2013-02-14

**Authors:** German L. Farfalli, Luis A. Aponte-Tinao, Miguel A. Ayerza, D. Luis Muscolo, Patrick J. Boland, Carol D. Morris, Edward A. Athanasian, John H. Healey

**Affiliations:** ^1^Department of Surgery, Orthopaedic Surgery Service, Memorial Sloan-Kettering Cancer Center, Weill Cornell Medical College, Cornell University, New York, NY, USA; ^2^Institute of Orthopedics “Carlos E. Ottolenghi,” Italian Hospital of Buenos Aires, Buenos Aires, Argentina

## Abstract

Allograft-prosthesis composite (APC) can restore capsular and ligamentous tissues of the knee sacrificed in a tumor extirpation. We asked if performing APC would restore knee stability and allow the use of nonconstrained arthroplasty while preventing aseptic loosening. We retrospectively compared 50 knee APCs performed with non-constrained revision knee prosthesis (Group 1) with 36 matched APCs performed with a constrained prosthesis (Group 2). In Group 1, the survival rate was 69% at five and 62% at ten years. Sixteen reconstructions were removed due to complications: eight deep infections, three fractures, two instabilities, one aseptic loosening, one local recurrence, and one nonunion. In Group 2, the survival rate was 80% at five and 53% at ten years. Nine reconstructions were removed: 3 due to deep infections, 3 to fractures, and 3 to aseptic loosening. In both groups, we observed more allograft fractures when the prosthetic stem does not bypass the host-donor osteotomy (*P* > 0.05). Both groups had mainly good or excellent MSTS functional results. Survival rate and functional scores and aseptic loosening were similar in both groups. A rotating-hinge APC is recommended when host-donor soft tissue reconstruction fails to restore knee instability. The use of a short prosthetic stem has a statistical relationship with APC fractures.

## 1. Introduction 

The potential benefits of allograft-prosthesis composite (APC) include restoration of bone stock, possible reattachment of tendons to the graft, and improved longevity through load-sharing properties of the allograft [1–3]. The ongoing challenge is choosing the most appropriate implant for this type of reconstruction. Constrained implants provide the needed stability for arthroplasty in the presence of a deficient soft-tissue envelope. However there is a requisite transmission of grater forces to the fixation interfaces, which may lead to premature aseptic loosening [4, 5]. A posterior-stabilized or semiconstrained revision knee arthroplasty is usually contraindicated in knees with severe metaphyseal bone loss and instability [3, 6, 7]. Nevertheless, when the less constrained device is combined with a massive allograft stabilizing soft tissue elements may be sufficient [8–10]. If stability is not maintained, problems of edge loading, aseptic loosening, and fracture may ensue. Each type of articulation has theoretic advantages and disadvantages.

The type of arthroplasty device to be used is determined by how much stability is lost from the tumor resection [8–10]. If a significant amount of the collateral ligaments is taken with the tumor, a constrained articulation, such as a rotating hinge, may be indicated. Nevertheless, if a minimum amount of soft tissue must be sacrificed or soft tissue reconstruction restores stability, a less constrained device, such as a constrained condylar knee, may potentially be chosen. Only small series of patients who have undergone these procedures have been reported [8–13], and the competing techniques have not been compared.

Given the lack of outcome data of using these kinds of reconstructions, we compared the experiences of similar patient populations at two orthopedic oncology centers utilizing either a constrained or nonconstrained prosthesis in patients treated with knee APC. We therefore determined (1) the overall APC survival and (2) the differences in survival between constrained and nonconstrained APCs, (1) to identify and compare complications associated with failure in each different group and (2) and to assess functional results.

## 2. Materials and Methods 

Between January 1989 and August 2008, we retrospectively reviewed 93 consecutive cases collected from two different Orthopaedic Oncology Services' databases. A minimum followup time of 2 years was required for inclusion, unless failure occurred earlier. We excluded 7 patients. Five of them died of disease before 2 years of followup and the remaining 2 cases were lost before 2 years of followup. The duration of followup was calculated from the surgery to the date that the patient was last seen (for asymptomatic patients) or the date of death, amputation, or revision surgery. This left 86 patients in the study.

 Patients were divided into two groups: those who received a nonconstrained APC (Group 1: 50 patients) and those who received a constrained APC (Group 2: 36 patients).

In Group 1 (nonconstrained APC), the reconstruction was indicated for a revision of another reconstruction in 26 cases, tumor resection in 22 cases, and traumatic bone loss in 2. Twenty-eight were distal femur and 22 proximal tibia APC reconstructions. Most of the distal femoral APCs were indicated for a fracture of osteoarticular allografts or when femoral attachments of the cruciate ligaments were involved by the tumor. Proximal tibia APCs were indicated mainly for extensor mechanism reconstruction in skeletally mature patients, when tibial attachment of the cruciate ligaments was involved by the tumor, and for resurfacing of a failed osteoarticular allograft. Thirteen patients in this group received chemotherapy. The average followup in Group 1 was 69 months (range, 8 to 141 months). Demographics data are shown in Table 1.

**Figure 1:**
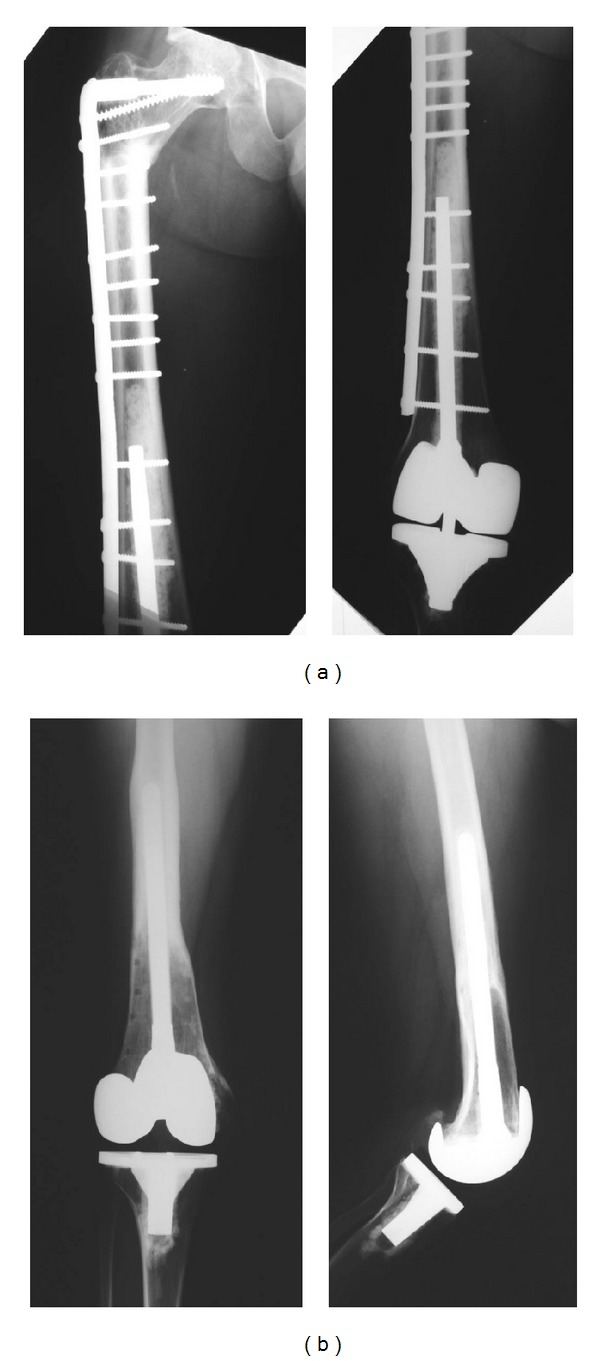
Reconstructions performed for distal femur in Group 1. (a) Distal femoral APC with a short stem. (b) Distal femoral APC with a long stem.

**Figure 2:**
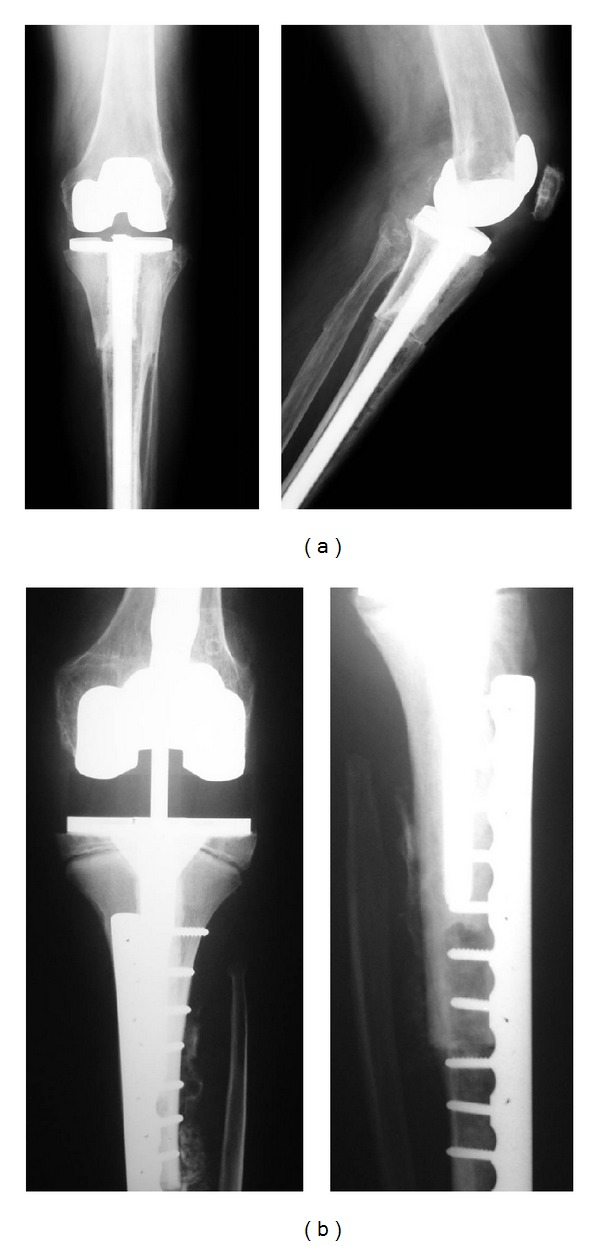
Reconstructions performed for proximal tibia in Group 1. (a) Proximal tibia APC with a long stem. (b) Proximal tibia APC with a short stem.

Surgical technique for Group 1 (Figures 1 and 2): through an extended anterior-medial approach, the tumor resection or the extraction of the previous reconstruction was made, preserving as much as possible the patient's soft tissue insertions. No extra-articular resection was performed in this group. All prostheses utilized in this group were nonconstrained modular revision prostheses. For APC fixations, different techniques were performed. In 23 patients the reconstruction consisted in prosthesis cementation in the allograft and implanted without cement in the residual tibial or femoral diaphysis without an osteosynthesis plate. In 27 patients a compression plate was placed to improve contact and stability at the host donor osteotomy. However, in 11 of 27 patients, a short stem that did not bypass the host-donor osteotomy was utilized, so fixation was only with the osteosynthesis plate. The corresponding component was cemented into the host bone on the opposite side of the joint. The prostheses utilized in this group were Coordinate Revision Knee System (DePuy, Warsaw, IN) in 11 cases, Scorpio TS Revision Implant (Stryker Orthopaedics, Mahwah, NJ) in 12, Sigma PFC Revision Implant (DePuy, Warsaw, IN) in 6, Next Gen LCCK Revision Implant (Zimmer, Warsaw, IN) in 6, Continuum Knee System (CKS) in 4 (Stratec Medical, Oberdorf, Switzerland), and Genesis II Implant (Smith & Nephew, Memphis, TN) in 11. 

Nonirradiated fresh-frozen allografts were used as previously described [15]. After the assembling of the allograft-prosthesis composites and the receiver, the host posterior capsule and the collateral ligaments were sutured to corresponding ligaments of the graft. In seven patients the medial collateral ligament was too short to be reattached, and in 2 patients the lateral collateral ligament also was too short to be reattached to the corresponding structure. In those patients almost the entire medial and lateral ligaments were replaced with the ligament provided by the allograft. In all the proximal tibial reconstructions, the extensor mechanism then was reconstructed to the corresponding tissue of the allograft using a previously described technique [16].

In Group 2 (constrained APC), the reconstruction was indicated for a revision of tumor endoprosthesis in 14 patients, revision of an osteoarticular allograft in three, and tumor resection in 19 cases. Most distal femoral APCs were indicated when the proximal femur was too narrow or short to receive an intramedullary stem. Proximal tibia APCs were indicated mainly in skeletally mature patients for extensor mechanism reconstruction. Fifteen patients received chemotherapy in this group. Nineteen were proximal tibia and 17 distal femoral APC reconstructions. The average followup in Group 2 was 75 months (range, 7 to 197 months). Demographics data are shown in Table 1. 

**Figure 3:**
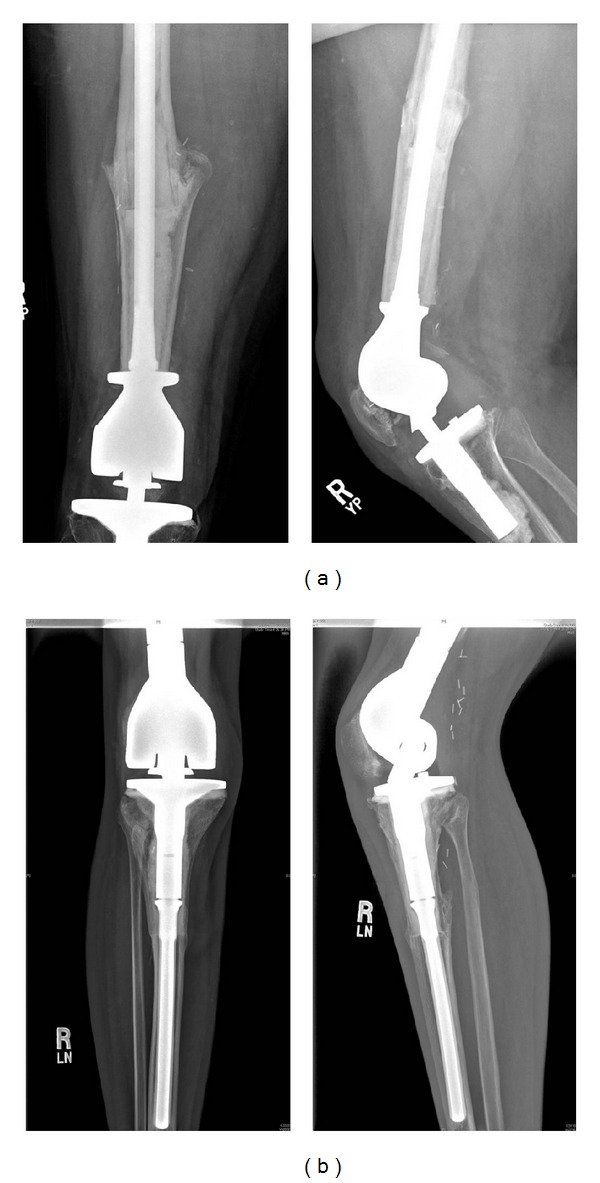
Standard reconstructions for Group 2. (a) Distal femoral APC. (b) Proximal tibia APC.

Surgical technique for Group 2 (Figure 3): through an extended anterior-medial or anterior-lateral approach, the tumor resection or extraction of the previous reconstruction was made. An extra-articular resection was performed in four patients and intra-articular resection in 32. The standard technique of reconstruction with the composite prosthesis involves the use of a rotating hinge revision modular prosthesis cemented in the allograft and implanted in the residual tibial or femoral diaphysis with cement in 22 cases or without cement in 14 cases. The standard technique of reconstruction with the composite prosthesis involves the use of a rotating hinge revision modular prosthesis (Finn Knee prostheses; Biomet, Warsaw, IN) cemented in the allograft and implanted in the residual tibial or femoral diaphysis with cement in 24 cases or without cement in 12 cases. Of this series of 36 patients, only two patients received another type of hinge prosthesis. One had a custom-made Lane-Burstein (Biomet, Warsaw, IN) prosthesis and the other a Guepar prosthesis (Wright Medical, Arlington, TN). Three cases of this group had a short stem that did not bypass the host-donor osteotomy, so dynamic compression plates stabilized the graft-host junction. 

Fresh-frozen nonirradiated allografts were used, and bacteriological and viral studies were performed in accordance with the recommendations of the American Association of Tissue Banks and the tests available at the time. Eleven patients were reconstructed with a telescope allograft technique [17].

After the assembling of the allograft-prosthesis composites and the host, the capsule and the collateral ligaments were sutured when possible. 

In all the proximal tibial reconstructions, the patellar tendon was repaired by direct suture overlapping the autologous proximal part onto the distal one provided by the graft, and a medial gastrocnemius rotation flap was performed in 16 of the 19 tibial reconstructions. 

The functional evaluation was performed in both groups using the revised 30-point functional classification system established by the Musculoskeletal Tumor Society [18].

Surgical complications were defined according to the Clavien-Dindo classification [19] that separates complications in five grades: Grade I, any deviation from the normal postoperative course without the need for pharmacologic treatment or surgical, endoscopic, and radiographic interventions, with acceptable therapeutic regimens including drugs, such as antiemetics, antipyretics, analgesics, diuretics, and electrolytes, and physiotherapy; Grade II, complication requiring pharmacologic treatment with drugs other than those allowed for Grade I complications; Grade III, complication requiring surgical, endoscopic, or radiographic intervention; Grade IV, life-threatening complication; and Grade V, death of a patient. We analyzed only Grades III, IV, and V complications in this series.

### 2.1. Statistical Analysis

 Survival of the different APCs was calculated using the Kaplan-Meier method. Differences in survival between groups were assessed with the log-rank test. To identify factors that affected the survival of the reconstructions a univariate analysis was carried out. A *P* value ≤0.05 was considered significant. We used SPSS 17.0 for Windows (Chicago, IL) for statistical analyses.

## 3. Results 

**Figure 4:**
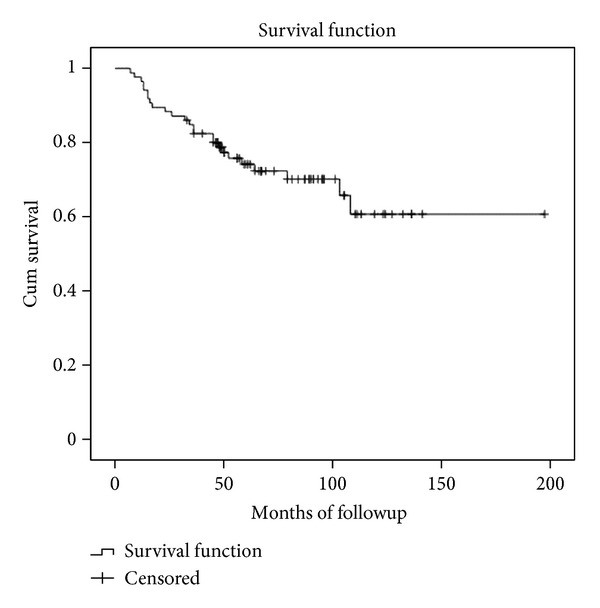
Kaplan-Meier curve showing the overall APC survival.

**Figure 5:**
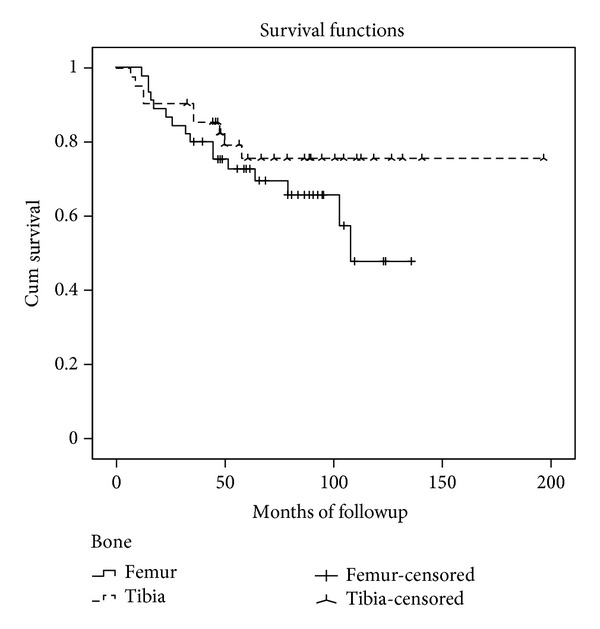
Kaplan-Meier curve showing the differences in APC survivorship according to the affected bone.

The overall APC survival was 70% at five years (SE 5.4%) and 61% (SE 7,8%) at 10 years. The mean APC duration was 140 months for all patients (SE 9.7, 95% confidence interval, 121 to 159 months). Distal femur APCs survival was 73% at five years (SE 6.8%) and 48% at ten years (SE 12.2%). The mean APC duration was 97 months for this location (SE 7.6, 95% confidence interval, 81 to 112 months). Proximal tibial APCs survival was 75% at five years (SE 7.3%) and ten years. The mean APC duration was 156 months for this location (SE 11.8, 95% confidence interval, 133 to 180 months) (Figures 4 and 5).

**Figure 6:**
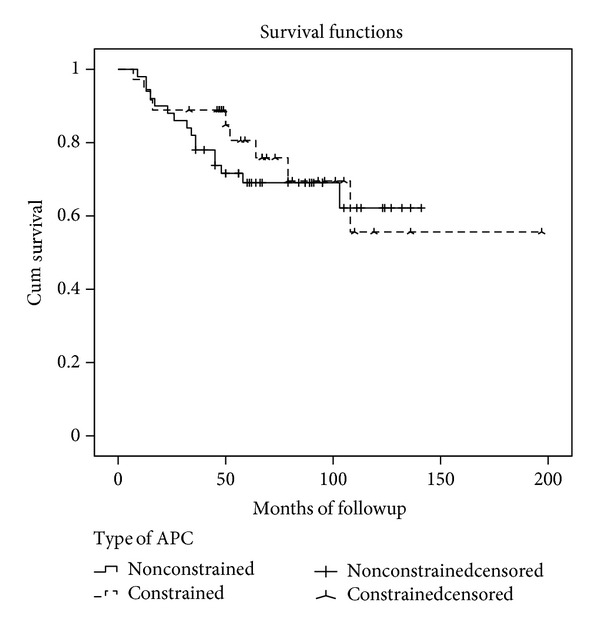
Kaplan-Meier curve showing the differences in APC survivorship between hinged and nonhinged knee replacements.

In Group 1 (nonconstrained APC), the survival rate was 69% at five years (SE 6.7%) and 62% at ten years (SE 8.9%). The mean APC duration was 104 months for all patients (SE 7,5, 95% confidence interval, 89 to 119 months) (Figure 6). In this group, four patients had a minor medial instability and four had major instability. Three of the minor ligament instability needed no external support, and the remaining patient used a cane. Two patients had a major medial instability, and they were revised with hinge prosthesis. The other two major instabilities refused second surgery and used an external brace. Thus the allograft ligamentous reconstructions restored stability in 42 of 50 patients.

Sixteen reconstructions were removed due to major complications: eight deep infections, three fractures, two instabilities, one aseptic loosening, one local recurrence, and one nonunion.

Of the 8 patients with a deep infection, four were proximal tibia reconstructions and four distal femoral APCs. In two patients, amputations were required due to a persistent infection. The remaining six patients with an infected allograft were treated with resection of the allografts-prosthesis and maintenance of limb length with an antibiotic-impregnated polymethylmethacrylate spacer. Antibiotics that were appropriate for the microorganisms that recovered from the site of the infected allograft prosthesis were administered for one to three months. After the infection was under control, a second limb-salvage procedure was performed in six patients. These included three knee endoprostheses, one new hinged APC, and two knee arthrodeses. 

The APC was removed in three patients with a fracture, endoprostheses applied in two patients, and a new APC placed in the remaining patient. Two allograft fractures were in distal femoral and one in proximal tibial APC. All fractures-occurred in patients with a short stem (Table 3). 

Both patients with a nonunion and with aseptic loosening were treated with a distal femoral endoprosthesis. The patient with a local recurrence was treated with an amputation.

Three other APC complications did not require removal of the allograft prosthesis including two peroneal nerve palsy (both after proximal tibial reconstruction) and one nonunion in a distal femoral reconstruction (treated with a new osteosynthesis and with autologous bone grafts).

Two patients died from tumor-related causes without APC failure after a two-year radiographic and functional followup was done.

For the patients who retained the APC (34 cases), the mean MSTS functional score at last followup was 25 of 30 (83.3%, range 10–30). For distal femoral APCs the mean score was 25 (83.3%, range 10–30) and 24.6 (82%, range 13–30) for proximal tibial APCs. Physical examination revealed that the arc of active motion of the knee averaged 94.4° (range, 45° to 120°). The mean extensor lag was 3.5° (range, 0° to 20°) (Table 4). 

In Group 2 (constrained APC), the survival rate was 80% at five years (SE 7,3%) and 53% at ten years (14,7%). The mean APC duration was 138 months for all patients (SE 17, 95% confidence interval, 105 to 171) (Figure 6). There were 12 complications among 36 patients of which 9 were major requiring removal of the APC and 3 were minor and could be solved while retaining APC. Nine reconstructions were removed: 3 due to deep infections, 3 to fractures, and 3 to aseptic loosening. 

Of the three patients with deep infection, all were distal femoral APCs. One of them had a previous reconstructive surgery. One of these patients was treated with resection of the APC and maintenance of limb length with an antibiotic-impregnated polymethylmethacrylate spacer. Several spacer exchanges were necessary to control the infection. Antibiotics appropriate for the microorganisms that recovered from the site of the infected APC were administered for several months. After the infection was under control, a total femur replacement was performed. In the remaining two patients an amputation was indicated due to persistent infection.

APC complications did not require us to remove the allograft prosthesis: one with polyethylene failure, one with superficial infection, and one with secondary reinforcement of the patellar tendon.

Of the three patients with fractures, in two, the APC was removed and a new APC applied, and in the remaining patient, an endoprosthesis was placed. Two allograft fractures were in distal femoral and one in proximal tibia APC. Two of them happened in patients with a short stem. The femoral fractures occurred in APCs performed with the Guepar and with the Lane-Burstein prostheses. The tibial fracture was in short-stem APC performed with Finn Knee prosthesis (Table 4).

The three patients with aseptic loosening were revised with a new APC in two patients and with a distal femoral endoprosthesis in one. In two patients, the failure occurred after five years of followup, and in the remaining one loosening happened at 19 months. All patients with aseptic loosening were distal femoral APC.

Five patients died from tumor-related causes without APC failure after a two-year radiographic and functional followup could be carried out.

The patients who retained the APC (27 cases), the mean MSTS functional score at last followup was 25.3 of 30 (84.3%, range 13–30). For distal femoral APCs the mean score was 26 (86.6%, range 21–29) and 24.9 (83%, range 13–30) for proximal tibial APCs. Physical examination revealed that the arc of active motion of the knee averaged 90° (range, 25° to 120°). The mean extensor lag was 8° (range, 0° to 70°). Two patients with extra-articular resection at the primary surgery showed the worst extension lag (50° and 70°) (Table 4). No patient had clinical instability.

Comparison of the two groups revealed that no statistical difference was observed in survival rates, functional scores, or number of complications and the incidence of aseptic loosening (Table 2). Group 1 showed a statistical relationship with residual knee instability (*P* = 0.034). In both groups, the use of a short prosthetic stem has a statistical relationship with APC fractures (*P* = 0.0001).

## 4. Discussion 

Allograft-prosthetic composite combines a metallic implant with a large fragment allograft to reconstruct bone and joint deficiency [8, 12, 13]. This procedure has several potential advantages. By combining a metallic implant with an allograft, the surgeon has the option of replacing as many bones as necessary. In addition, by resurfacing the bone with an implant, allograft cartilage degeneration is not a problem. The composite allograft also affords the opportunity for soft tissue attachment, thus making joint stability and functional recovery potentially greater [2, 13, 20]. 

We acknowledge some limitations of this study. This is a retrospective study with a relatively small number of patients with intermediate average followup, and thus it had limited ability to detect potential long-term differences between the groups, including survival and rates of individual complications. Given the relative rarity of this reconstructive problem and the unique surgical treatment for each individual, it would be difficult to obtain a longer series for more robust results. However, to our knowledge, this is the largest comparative study of alternative methods of knee APCs. Despite these limitations we could see some meaningful trends.

The overall APC survival was 70% at five years (SE 5.4%) and 61% (SE 7,8%) at 10 years. Distal femur APCs survival was 73% at five years (SE 6.8%) and 48% at ten years (SE 12.2%). Proximal tibial APCs survival was 75% at five years (SE 7.3%) and ten years. These survival rates are similar to those of other reconstructive techniques such as endoprosthesis or knee osteoarticular allograft [13, 14, 21–25]. Previous reports showed a worse survival rate in proximal tibial compared to distal femur reconstructions [11, 14, 21, 22, 24, 26]; nevertheless in this series, proximal tibial had a slightly better performance than distal femur APC reconstructions. No difference was observed in survival rates between both groups at five years. 

In Group 1, infection was the main cause of failure. As reported in the literature, infection rate could be related to multifactorial causes. Common factors associated with infection in similar reconstructions include an extensive soft tissue dissection, compromised vascular supply, proximal tibial location, the immunosuppressive effect of chemotherapy, a long operating time, blood transfusion, and obesity [27]. However, the rates were not higher than those reported by other groups with just an osteoarticular allograft or endoprosthetic alone over the same time period [24, 26, 28, 29].

Aseptic loosening was observed in both groups, but was more frequent in the hinged APCs (3 cases: 8% versus 1 case: 2%), but these differences were not statistically significant. These failures may be related to the stress concentration at the stem-bone junction of hinged implants [4, 22, 23]. The use of a rotating-hinge mechanism may decrease torque transmitted to the implant interfaces [4, 6] but the forces still exceed those of a nonhinged prosthesis. 

Instability was observed in nonhinged APCs. Of the six patients with instability, four of them had a minor medial instability and two patients had a major medial instability with indicatioan for revision surgery. In situations like extra-articular resections or massive resections of host ligaments, rotation hinged APCs can provide initial stability [10]; therefore nonhinged APCs should be avoided. 

Several factors may have played a role in occurrence of allograft fracture: irradiation of the allografts [30], perforation of the cortex [31], and nonunion [32]. We found that utilization of a short stem was a risk factor for allograft failures in both groups even when an external plate was placed for APC fixation. Proximal tibia and distal femur allografts are very wide with a thin cortex at the metaphyseal level. Therefore, stress forces localized at the tip of the stem could fracture the APC even when an external plate is placed to support the allograft. Also, in Group 2, two of the fractures occurred in the more constrained prosthesis (Guepar and Lane-Burstein), and the only long-stem fracture was with the Lane-Burstein APC (Table 4). 

The MSTS functional scores in our study were similar to similar reconstructive option [4, 8, 13, 14, 21–23]. 

In this study, survival rate and functional scores were similar in both groups. Aseptic loosening may have been lower in APC performed with a nonhinged prosthesis, but rotating-hinge APC is recommended when host-donor soft tissues reconstruction is insufficient to reestablish stability. The use of a short prosthetic stem that does not bypass the host-donor osteotomy has a statistical relationship with APC fractures.

## Figures and Tables

**Table 1:** Differences between groups of APC knee replacements.

	Group 1 (nonconstrained APC)	Group 2 (constrained APC)
Number	50 patients	36 patients
Diagnostic (*n*)	Revision, osteoarticular allograft (20) Revision, knee prosthesis (6) Osteogenic sarcoma (8) Chondrosarcoma (7) Leiomyosarcoma (2) Fibrosarcoma (2) Giant cell tumor (2) Desmoplastic fibroma (1) Traumatic bone loss (2)	Revision, tumor endoprosthesis (14) Osteogenic sarcoma (11) Revision, osteoarticular allograft (3) Chondrosarcoma (3) Lymphoma (2) Soft tissue sarcoma (1) Malignant fibrohistiocytoma (1) Giant cell tumor (1)
Followup	69 months (range, 8–141 months)	75 months (range, 7–197 months)
Age	35 years old (range, 15–80)	35 years old (range, 8–84)
Sex	22 females; 28 males	18 females; 18 males
Location (*n*)	Distal femur (28) Proximal tibia (22)	Distal femur (17) Proximal tibia (19)

**Table 2:** Comparison of the results between groups.

	Group 1 (nonconstrained APC)	Group 2 (constrained APC)
Survival rate at 5–10 years	69%–62%	80%–53%
Failures	16 cases	9 cases
Cause of revision (*n*)	Deep infection (8) APC fracture (3) Instability (2) Nonunion (1) Aseptic loosening (1) Local recurrence (1)	Aseptic loosening (3) APC fracture (3) Deep infection (3)
Instability	8 cases	No cases

**Table 3:** Features of patients with fractures.

	Group 1 (nonconstrained APC)	Group 2 (constrained APC)
Fractures (*n*)	3	3
Stem	3 short stems	2 short stems,1 long stem
Prosthesis*	1 Coor, 1 PFC, 1 SN	1 Guepar, 1 LB (ls), 1 Finn
Plate fixation	All cases	2 cases (Guepar and Finn)
Stem fixation	None	1 case (LB ls)

Coor: Coordinate prosthesis; PFC: Johnson & Johnson prosthesis; SN: Smith & Nephew prosthesis; LB (ls): Lane-Burstein long stem.

**Table 4:** Comparison of mean functional scores between groups (Musculoskeletal Tumor Society).

Measure	Group 1 (nonconstrained APC)	Group 2 (constrained APC)
Pain	4.5	4.5
Function	3.8	3.7
Acceptance	4.4	4.5
Supports	4	4.1
Walking	4.3	4.4
Gait	3.9	3.9
Total score	25 (83.3%)	25.3 (84.3%)
Range of motion	94° (45° to 120°)	90° (25° to 120°)
Extensor lag	3.5° (0° to 20°)	8° (0° to 70°)
